# Explosive Fibonacci-sequence growth into unusual sector-face morphology in poly(l-lactic acid) crystallized with polymeric diluents

**DOI:** 10.1038/s41598-020-67567-5

**Published:** 2020-07-02

**Authors:** Graecia Lugito, Selvaraj Nagarajan, Eamor M. Woo

**Affiliations:** 1 Department of Chemical Engineering, National Cheng Kung University, No. 1 University Road, Tainan, 70101 Taiwan; 2 Department of Chemical Engineering, Institute Technology Bandung, Jl. Ganesha 10, Bandung, 40132 Indonesia

**Keywords:** Biomaterials, Soft materials

## Abstract

Lamellar assembly in unusual sector-face PLLA spherulites from crystallization of poly(l-lactic acid) (PLLA) diluted with amorphous poly(methyl methacrylate) (PMMA). The growth and morphology of the crystalline structures is studied using polarized optical microscopy (POM), atomic-force and scanning electron microscopies (AFM, SEM). Crystals are also analyzed using differential scanning calorimetry (DSC) and small-angle X-ray scattering (SAXS). The two alternate sectored faces differ dramatically in their optical birefringence and top-surface and interior lamellar assembly. By originating from the nucleus center, an explosive fan-like sector of high-birefringence lamellae is packed by fractal growth from an initial single stalk into hundreds of branches upon reaching the periphery, with the number of stalks increasing roughly by the Fibonacci sequence along the radial distance. The exploded pattern resembles a cross-hatch grating structure, and displays a cauliflower-like fractal-branching of optical birefringence blue/orange stripes. This finding suggests that growth with periodic branching is one of the main mechanisms to fill the ever-expanding space in the spherulitic 3D aggregates.

## Introduction

Crystal aggregation upon crystallization leads to formation of circular entities commonly known as spherulites. Owing to that fact that nucleation and growth routes are influenced by complex interplays between kinetics and thermodynamics factors, lamellar assembly can vary. Multiple-face morphologies on crystallized sample have been found in polymorphic polymers and were regarded to be associated with the polymorphism^1–9^. Several terminologies such as concomitant crystallization, cross nucleation, epitaxial growth have been widely used to explain the phenomena. However, the mechanisms of formation of such multiple morphologies have not yet been fully expounded and further investigations are needed in order to comprehend the complex phenomena in hierarchically structured spherulites^9^. Normally a spherulite is uniform in its optical birefringence as the lamellae, indistinguishable among them, usually grow and radiate outward from a common nucleus center. Dual-face or multiple-face (i.e., sectored into wheel-like) morphologies packed into a single polymer spherulite may be puzzling. Such peculiar spherulites with sector-combined morphologies have been discovered in crystallization of several polymers in neat as well as in blends with another compound^10–15^. Crystallization of poly(l-lactic acid) (PLLA) in presence of amorphous poly(vinyl-phenol) (PVPh) or atactic poly(methyl-methacrylate) (aPMMA), for examples, results in formation of three dramatically different types of spherulitic morphologies with type-3 spherulites being a half-and-half combination of type-1 and type-2 ones^14,15^—termed as “sector-face” spherulites. The eye-like-face is composed of flat pebble-like lamellae appearing as an optically low-birefringent region; the other face consists of fibrous dendritic lamellae, with fractal tree-branch-like growth, appearing as an optically high-birefringent region^16^. Deeper analyses to the interior lamellar assembly of these spherulites might elucidate why and how the PLLA spherulites could have displayed such complex morphology behavior when crystallized in presence of amorphous aPMMA.

A couple of earlier work^16,17^ have revealed interesting dependence of PLLA morphology on crystallization temperature (T_c_), PLLA/diluent compositions, as well as rigid top-cover glass and thickness < 5 μm constraint on specimens during crystallization, etc. Without any top-surface restriction on specimens during crystallization, neat PLLA exhibits negative-birefringent spherulites regardless of T_c_. PLLA crystallized with aPMMA as a diluent, however, displays dramatic morphological changes with regard to composition and T_c_. Increase in PMMA content, as well as T_c_, gradually transforms the spherulitic morphology of the crystallized PLLA spherulites from a negative type with strong birefringence into a positive-type with weak birefringence. Most notably, appearance of a sector-face morphology (spherulites with dual faces originating from a common nucleus center) in PLLA/PMMA = 80/20 blend when crystallized at T_c_ = 100–120 °C. The reversion of optical types from negative to positive birefringence suggests that the crystal axes in these two types (i.e., two faces) are reverted in the sector-face morphology. The dual faces (termed as “sector-face” or “Janus-face” with two opposed faces) with the strong-birefringence dendrites in one face and texture-less crystals as low-birefringence face are puzzling and interesting. The increasing sample thickness to 20 or 30 μm hinders the formation of such peculiar spherulites. Peculiar “Janus-face” or “sector-face” PLLA spherulites with a combination of two contrast morphologies and optical birefringence patterns have been reported in an earlier study^16,17^, in which the two opposed faces of spherulites contain directionally anisotropic dendrites of unusually high birefringence in one sheaf-shape face and dot-like crystals (low optical birefringence) of extremely low birefringence in the other bivalve-shape face (or eye-like regions), which had been investigated for detailed mechanisms of lamellae assembly.

**Figure 1:**
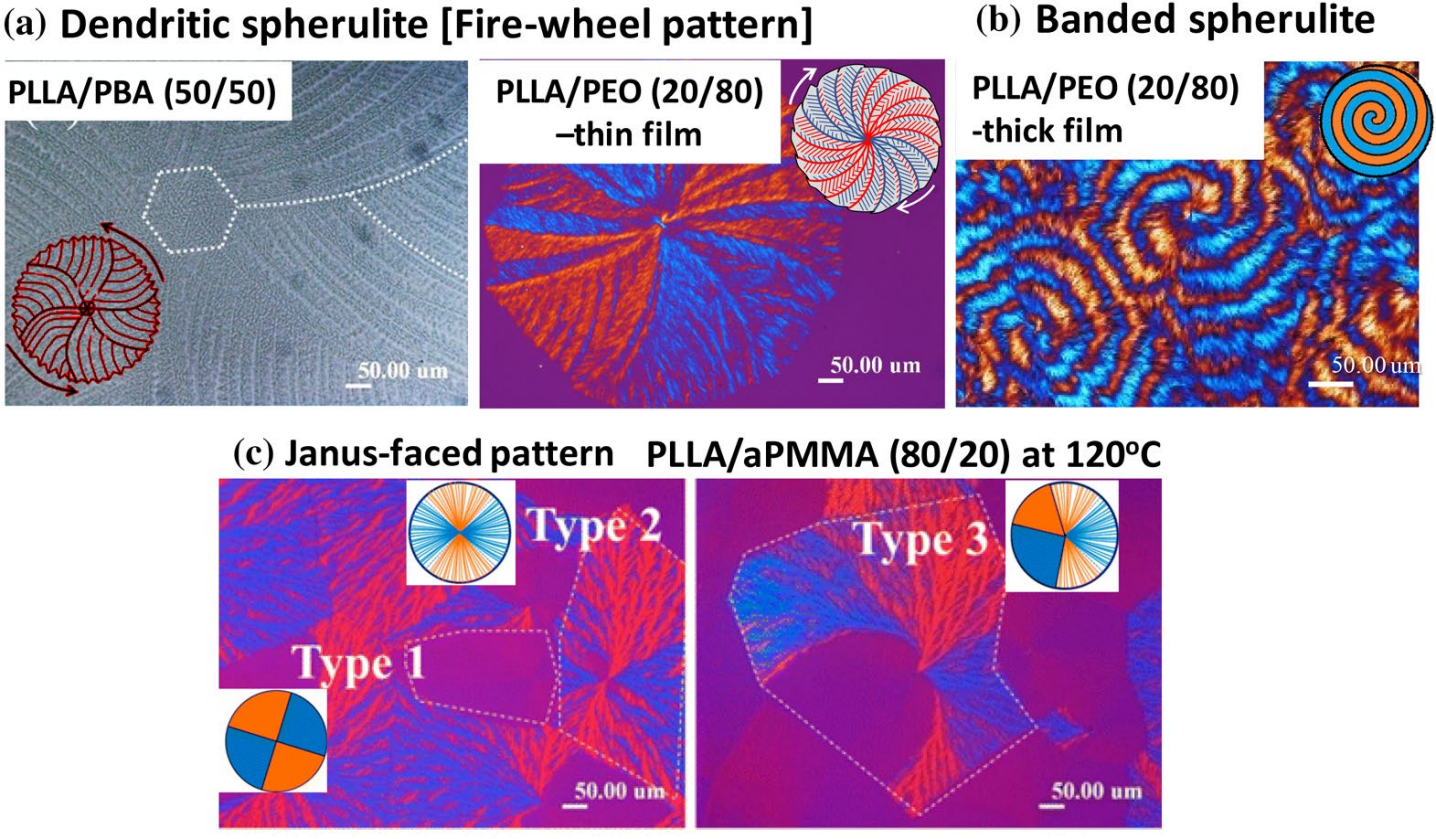
Representative morphologies by crystallization of poly(l-lactic acid) in various PLLA/diluent mixtures: (**a**) Fire-wheel dendritic^14^, (**b**) ring-banded type, reprinted with permission from Ref.^18^, and (**c**) Janus-face composite type (half and half dendritic/ringless)^15^. All images in this scheme are reprinted with copyright permissions from cited Refs.

To summarize for visual appreciation, Fig. 1 is illustrated here to list many of the diversified morphology patterns by crystallization of poly(l-lactic acid) in various PLLA/diluent mixtures, including the fire-wheel dendritic pattern in PLLA/PBA and thin-film PLLA/PEO, (b) ring-banded type in thick-film PLLA/PEO, and (c) Janus-face composite type (dendritic + ringless) in PLLA/aPMMA. The factors of growth kinetics leading to Type 1, Type 2, Type 3 PLLA spherulites in Fig. 1c have been discussed in an earlier paper^15^. In general, Type 1 PLLA (low-birefringence) is more prone to occur in thin-film specimens, while Type 2 dendritic type (high birefringence) is more likely in thick-film specimens. That is, there may be some local variation of film thickness in a same specimen, where some dendritic spherulites grow faster to drain off the neighboring regions. In Type 3 (Janus-face) PLLA spherulite, it is because the dendritic face (high-birefringence) grows much faster (from a common nucleus sheaf-bundle), leading to a bi-valve region (low-birefringence) being drained off the molten species to become a low-birefringence face.

**Figure 2:**
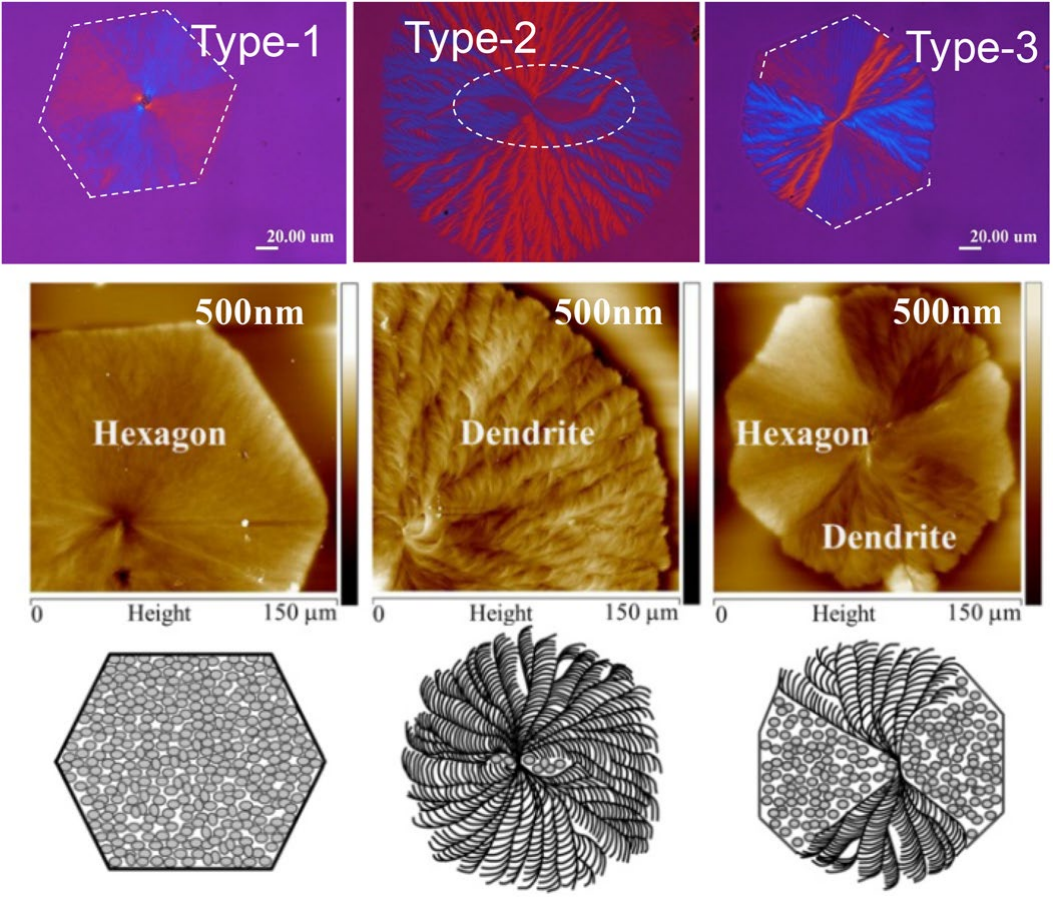
Simultaneous co-existence of multiple types of PLLA spherulites crystallized from PLLA/PVPh (70/30) blend at T_c_ = 120 °C. Type-1: sole hexagon; Type-2: sole dendritic pattern; Type-3: Janus-face with hexagon/dendritic patterns combined in a same spherulite. Re-printed with permission from cited Ref.^19^.

In addition, in a same polymer specimen crystallized at a same controlled condition, multiple types of spherulites differing dramatically in their optical birefringence patterns and lamellar assemblies, up to three or four types, may be found to co-exist. Such cases can be exemplified in PLLA diluted by H-bond interacting poly(p-vinyl phenol) (PVPh), crystallized at T_c_ = 120 °C^19^. Figure 2 shows that three dramatically different types of PLLA spherulites are simultaneously present in PLLA/PVPh (70/30) at T_c_ = 120 °C, where Type-1 is a sole hexagon-pattern filled with dot-like grainy crystals; Type-2 is a sole dendritic pattern packed with fern-leaf-like fibrous lamellae/branches; Type-3 is Janus-face with hexagon/dendritic patterns combined in a same spherulite sharing the same nucleus.

Variety of PLLA spherulites suggests that several complicated kinetic factors may have led to such results. Dendrites in such a sectored portion of the PLLA spherulites are made of explosive fan-like branches, in fractal growth cycles, evolving from a common central nucleus. The objective of study was to further elaborate plausible mechanisms of dual-face morphologies, composed of sectored dendrites, in the same PLLA spherulite crystallized from PLLA/aPMMA blends by microscopic analyses on detailed top-surface-relief morphologies.

## Results and discussion

**Figure 3:**
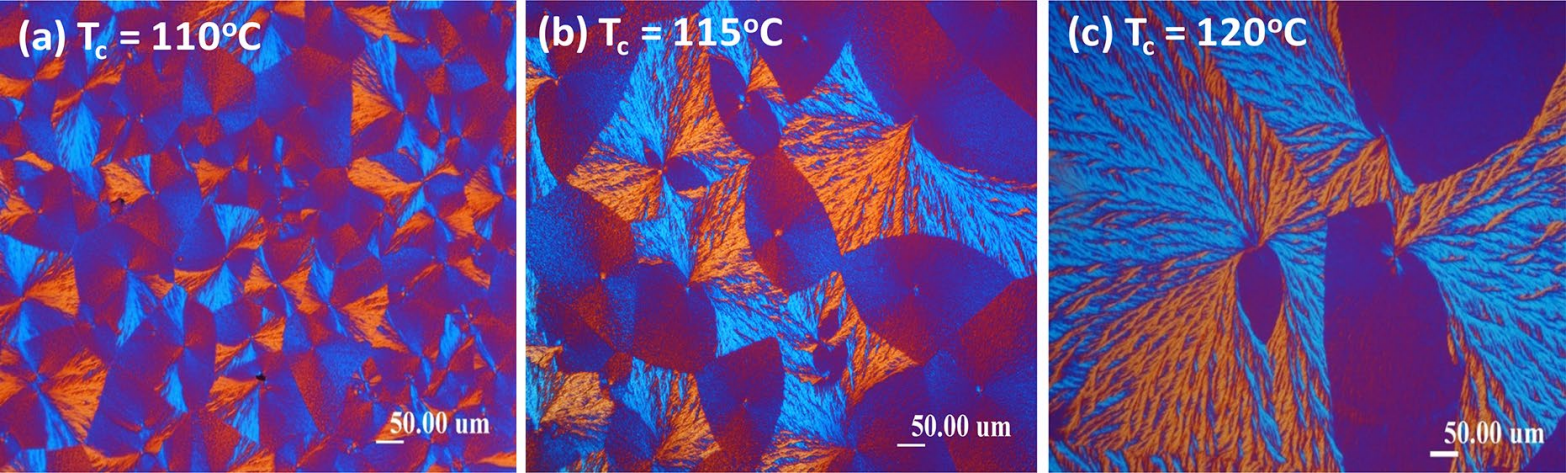
POM images of PLLA/PMMA (80/20) samples of a fixed film thicknesses ≈ 10 μm, melt-crystallized at various *T*_*c*_’s: (**a**) 110 °C, (**b**) 115 °C, and (**c**) 120 °C, as labeled.

Neat PLLA (M_w_ = 11 k) does not form dendritic spherulites at T_c_ = 115–120 °C. PLLA is blended with an amorphous diluent such as PMMA at specific mixture compositions and then crystallized, a peculiar phenomenon in morphology transformation is observed that might have been effected by the diluents. As shown in Fig. 3, only when it is mixed with proper contents of amorphous PMMA of a suitable M_w_ (240 k) would it crystallize into an unusual morphology pattern packed with several “sector-face” divisions in the spherulites, meaning more than two dramatically different patterns of lamellar co-assemblies aggregating in a spherulite. The two or more faces can be sectored into several divisions or in two opposed half-and-half divisions. In the latter case, the PLLA spherulite is specifically labeled as “Janus-face”; alternatively, if in several sector-like divisions, the spherulite is labeled as “sector-face”. Objective of blending 20 wt% amorphous PMMA into PLLA (PLLA/PMMA = 80/20 wt ratio) and crystallization at specific T_c_’s is specified as following. Amorphous PMMA, miscible with PLLA, was used as an effective diluent to induce unique “sector-face” PLLA morphologies with half-and-half composite birefringence patterns on spherulites, whose respective interior crystal assembly in these two faces was the main subject of this study. It is noted here that 80 wt% PLLA with 20 wt% PMMA in the blend was found to be the most effective for inducing the dendrite face of high optical birefringence, which constitutes a unique half-face of the PLLA spherulite with another half-face being a texture-less eye-like region (bivalve-shape) of low-birefringence retardation.

**Figure 4:**
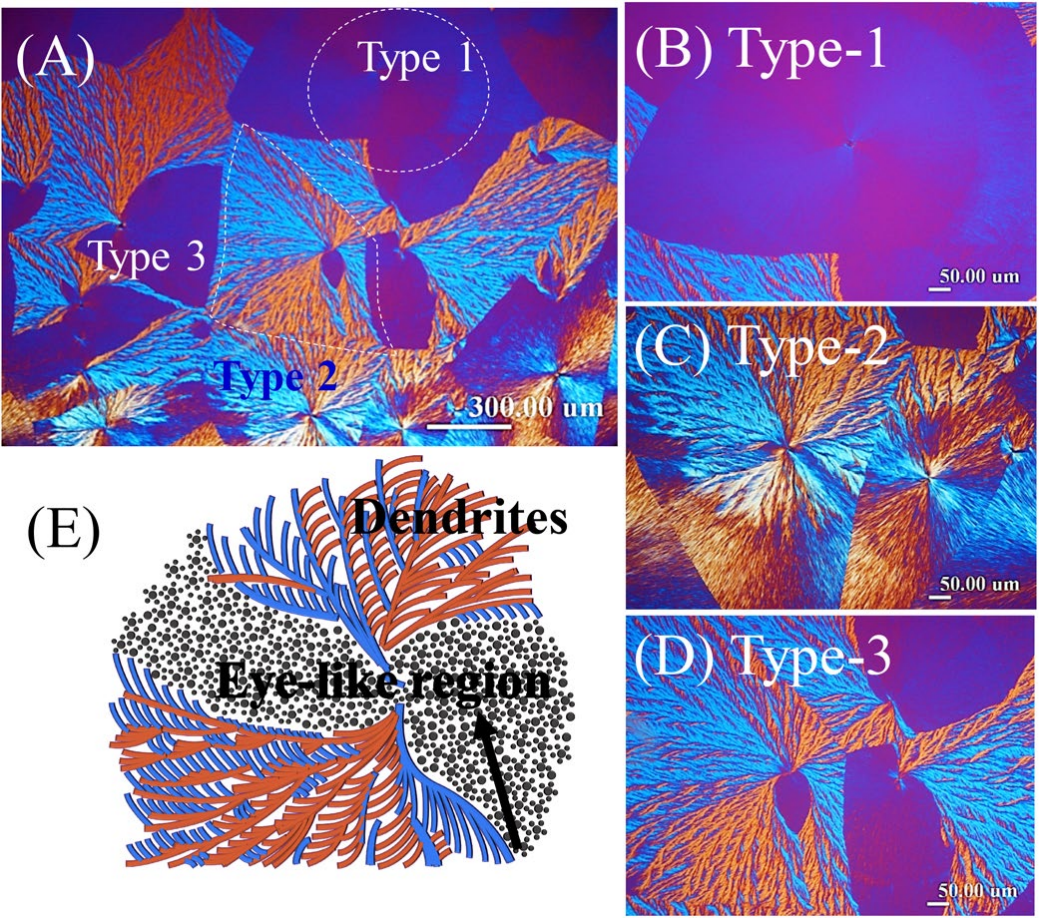
POM micrographs for diversified morphologies in PLLA spherulites crystallized from PLLA/PMMA (80/20) blend at T_c_ = 120 °C: (**A**) entire specimen at low magnification, and three distinct types of PLLA spherulites: (**B**) Type-1 positive-type low-birefringence, (**C**) Type-2 negative-type fully dendritic, and (**D**) Type-3 sector-face high-birefringence spherulites, and (**E**) detailed scheme for sector-face PLLA spherulite.

Figure 4A–D shows POM micrographs to cover a large area of specimen at lower magnification of PLLA spherulites crystallized from PLLA/PMMA (80/20) blend at 120 °C, where three different types of PLLA spherulites are clearly identified, which are labeled as: (Type-1) positive-type of low birefringence, (Type-2) negative-type dendritic, and (Type-3) “sector-face” spherulites. The “sector-face” PLLA spherulites are characteristic with a half-and-half combination of two contrast morphologies and optical birefringence patterns: low-birefringence positive-type portion (Type-1) and high-birefringence negative-type dendritic portion (Type-2). That is to say, the “sector-face” PLLA spherulites (Type-3) are composed of two different types of lamellar species of optically low-birefringence positive-type and high-birefringence negative-type dendrites, respectively. The scheme in Fig. 4E more vividly illustrates the features of lamellar assembly of two dramatically different structures collectively contributing to the sector-face composite characteristics. Note the two faces in the “sector-face” spherulites are not a result of incidentally overlapped stacking of two individual spherulites; they truly share a same nuclei center and evolve outward independently. An earlier investigation^16^ has pointed out that in the high-birefringence face of the “sector-face” PLLA spherulite, the dendrites predominantly evolve from two ends of sheaf-nuclei and fan out with multiple branches to fill the entire half of the “sector-face” PLLA spherulite. Perpendicular to the sheaf-nuclei direction is the low-birefringence face of the other half, which appears as a bivalve-shape pair of eyes (i.e., eye-like region) surrounded by the optically dominant dendrites. Thus, when crystallized at T_c_ = 120 °C on specimens of PLLA/PMMA blend, three different types of PLLA spherulitic morphology could simultaneously exist in the same PLLA spherulite. Note that PLLA/PMMA (80/20) specimen crystallized at T_c_ = 120 °C is similar to that at T_c_ = 115 °C as reported in an earlier work^16^; however, the relative fractions of Type-1, -2, and -3 vary sensitively with T_c_, although the “sector-face” morphology in the T_c_ range remains much the same.

It has been known that the molecular weight of aPMMA, film thickness of specimens, composition, and T_c_ all could affect the formation of sector-face morphology of PLLA in the PLLA/aPMMA blends. In addition, M_w_ of either PLLA or PMMA also affects the morphology. Effects of molecular weights (M_w_), blend compositions, as well as crystallization temperatures (T_c_) on PLLA crystal morphologies appearing during melt-crystallization of PLLA/aPMMA blends have been discussed in a previous publication^16^. For examples, blending high molecular weight spices of PMMA (240 k) and PLLA (51.6 k) promotes the formation of dual-morphological spherulites, yet increases the nucleation density of the samples. Oppositely, blending 11 k-PLLA with low-M_w_ PMMA reduces nucleation density, and when the M_w_ of PMMA is as low as 13 k, a typical ring-banded spherulite of PLLA appears, instead of the dual-morphology dendritic spherulite. In addition, an earlier study^16^ has pointed out that apparently, the sector-face spherulites initially grow by initiating the dendritic lamellae originating from the high-birefringent nucleation crystals and the growth of the dendritic lamellae can finally cover partially or entirely the low-birefringent crystals. Due to the faster radial growth of high-birefringent regions, the low-birefringent regions sometimes are overwhelmed and become much reduced into a narrow twin-eye-like area. There are two driving forces for leading to entirely high-birefringence negative-type dendritic PLLA spherulites (Type-2) or entirely low-birefringence positive-type PLLA spherulites (Type-1). Propositions of mechanisms are: (1) the dendritic lamellae have stronger tendency to grow in two different axes or forms, or (2) there are PLLA–PMMA molecular interactions (degree of entanglement) promoting the lamellae to grow in different axes or forms to become positive-type low birefringence PLLA spherulites. As these two forces are compromised in between, a sector-face PLLA morphology is resulted. Radial-oriented main lamellae with branching of platelet lamellae into perpendicularly grating structures are often observed in crystallization of PLLA in PLLA-diluent blends; however, the nature of the diluent, with different interacting intimacy with PLLA chains, may govern the growth and assembly of the lamellae in final spherulites. For PLLA crystallized with more strongly interacting diluents, such as ionic liquids or poly(p-vinyl phenol) (PVPh), the spherulitic PLLA morphologies are also different from those in the PLLA/PMMA blends, but no sector-face PLLA spherulites were ever found in either PLLA/ionic liquids or PLLA/PVPh blends^14,20,21^. These cases demonstrate the effects of diluents and film thickness, as well as T_c_ of course, on the lamellar assembly patterns in PLLA spherulites.

**Figure 5:**
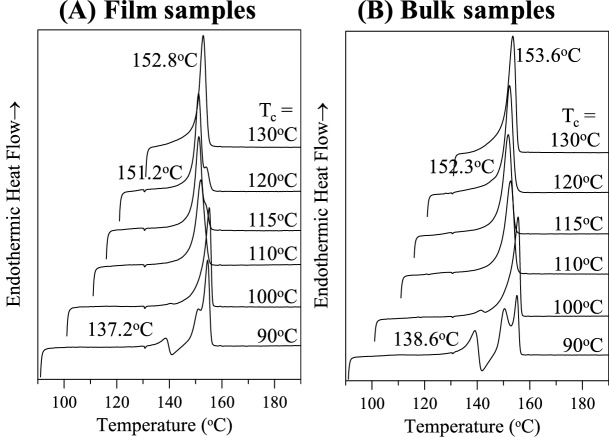
DSC curves of of variously crystallized 11 k-PLLA/240 k-aPMMA (80/20) of (**A**) thin films vs. (**B**) bulk specimens, thermal treatment: 190 °C-2 min-T_c_.

The thermal behavior of each lamellar types was then investigated using DSC (at a heating rate of 10 °C/min) and results are shown in Fig. 5. As a typical feature of the crystals crystallized at same temperatures, the dual morphologies in a same spherulite disappeared simultaneously (due to melting) around the same temperature (152 °C). To check the reproducibility and repeatability of the dual-morphological spherulite forms, in-situ POM observation was conducted on samples repeatedly melt-crystallized on a hot stage (programmed cooling/heating rate at 150 °C/min). The thermal treatment indicates that the dual morphologies in the sector-face PLLA spherulites have closely-spaced dual melting points (i.e., doublet peaks) with minor distinguishable differences (3–5 °C), suggesting that the basic lamellar thickness may differ a bit but not much. The lamellae in each faces of spherulites may aggregate into varieties of geometric shapes and dimension of bundles in these two faces as triggered by variation of kinetics driving forces.

**Figure 6:**
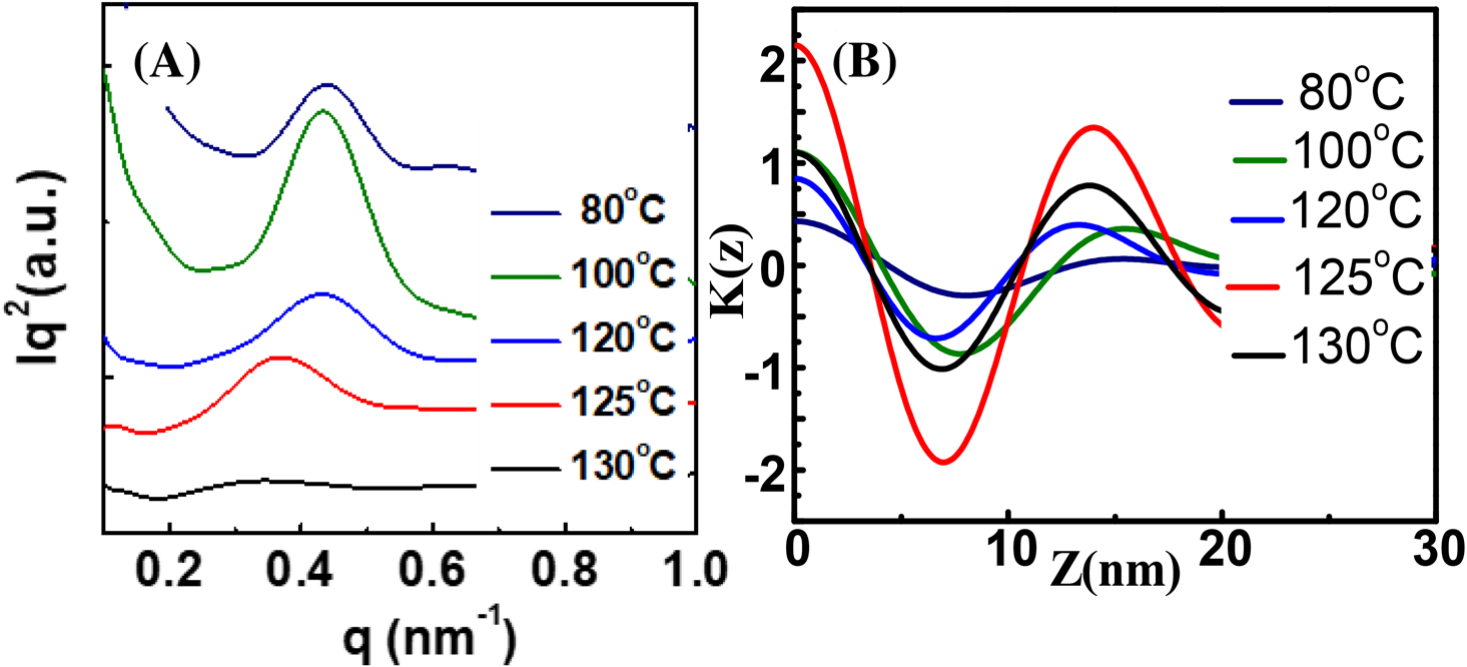
(**A**) 1D-SAXS results for the lamellae variation in PLLA spherulite of PLLA/PMMA (80/20) crystallized at T_c_ = 80 °C, 100 °C, 120 °C, 125 °C, and 130 °C, respectively, and (**B**) 1-D electron density correlation function estimated from SAXS data. (sample thickness = 5 µm).

The average lamellar thickness and its variation of PLLA/PMMA spherulites at different T_c_ were estimated using SAXS analysis, as shown in Fig. 6. Using a correlation function, the long period (L_o_), crystal thickness (L_c_), and amorphous thickness (L_a_) were estimated from 1D-SAXS plot. The crystallinity of the spherulite is calculated with X_c_ (%) = L_c_/L_o_*100. Increasing T_c_ from 80 to 120 °C slightly enhances the crystallinity of the spherulite from 57 to 60%. The PLLA/PMMA (80/20) specimens crystallized at or above 120 °C show crystallinity being stabilized at 59%. When the PLLA/PMMA (80/20) specimens are crystallized at T_c_ from 80 to 120 °C, respectively, they show transition covering three types of PLLA spherulites: (1) low-birefringence positive-type, (2) high-birefringence negative-type dendrites, and (3) sector-face complex composed of the former two types. For brevity, the details of normalized 1D-electron density correlation function K(z), along with the estimated values of lamellar thickness, are given in supporting information (ESI) Table S1. From the SAXS analysis, the three PLLA spherulitic morphologies (Type-1, 2, 3) do not show major differences in either lamellar thickness or crystallinity.

**Figure 7:**
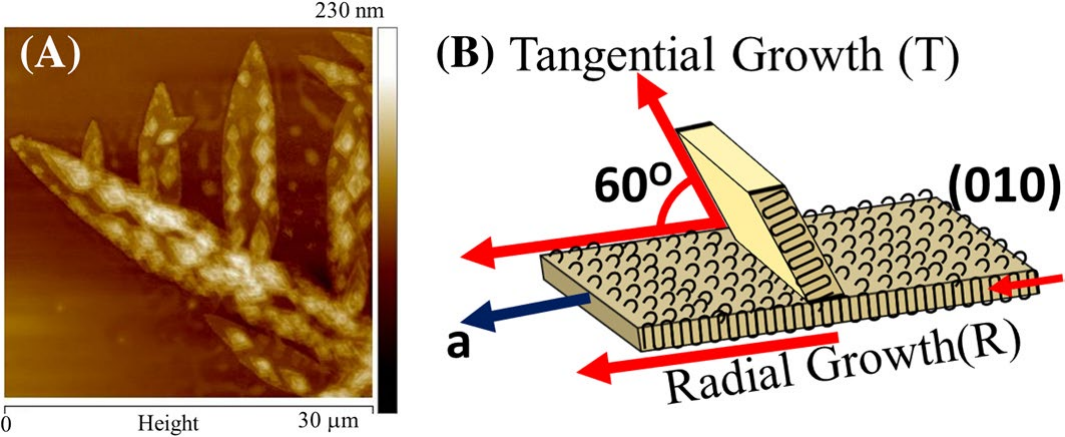
Representation of branching formation: (**A**) AFM images for lamellar branching at a specific angle (60° for PLLA) in branching, reprinted with permission from^22^, and (**B**) scheme for intersection angle in cross-hatch lamellae. Schemes re-drawn from Ref.^23^.

Note that outside the fractal-growth dendrites, the eyelike region is filled with smooth texture-less lamellae, which actually are packed by flat-on pebble-like crystals appearing as flattened regions when viewed at much larger magnifications^16^. It has been earlier reported that these flat “pebble-like” crystals on the top surface of eye-like region are actually the terminal ends of fibrous thin rod-like lamellae of nanometer diameters positioned at slant angles protruding from interior. Lamellae in these two faces are of entirely geometrical structures. One sector-face is packed with low-birefringence crystals with no apparent orientation on top surface, and the other face of the spherulite is packed with cross-hatch and highly dendritic lamellae that evolve like a fan from a tiny spot on the nucleus center. Figure 7 shows (A) AFM image and (B) schematics of lamellar branches mutually intersecting at angle of ~ 60° between the stalks and branches in the AFM image for branching growth in PLLA lamellae^22^. Such branching with cross-hatch lamellae with an intersection angle is also reported in iPP spherulite^23^ by Keller et al.^23^, who in studying the melt-crystallization of classical iPP, referred such lamellar morphology as an inter-woven structure made of “cross-hatch” of radial- and tangential-growth lamellae; however, note that for iPP, the entire spherulites are of the same uniform morphology with no sector-faces. The demonstrated lamellar assembly in iPP^23^ is used here for illustrating that thin, narrow fiber-like PLLA lamellae may emerge from a tiny cavity of the nucleus region, where a single tiny strand of fiber-like nuclei suddenly splays out like explosion into a massive fan-like grating structure. The AFM images of Fig. 7a clearly prove that the branching lamellae in PLLA are mostly packed with diamond-shape single crystals (orthorhombic lattices) that evolve by orienting from its crystal angle of ca. 60°. PLLA single crystals have an orthorhombic lattice with axes *a* = 10.66 Å, *b* = 6.16 Å, and *c* = 28.88 Å^24^.

**Figure 8:**
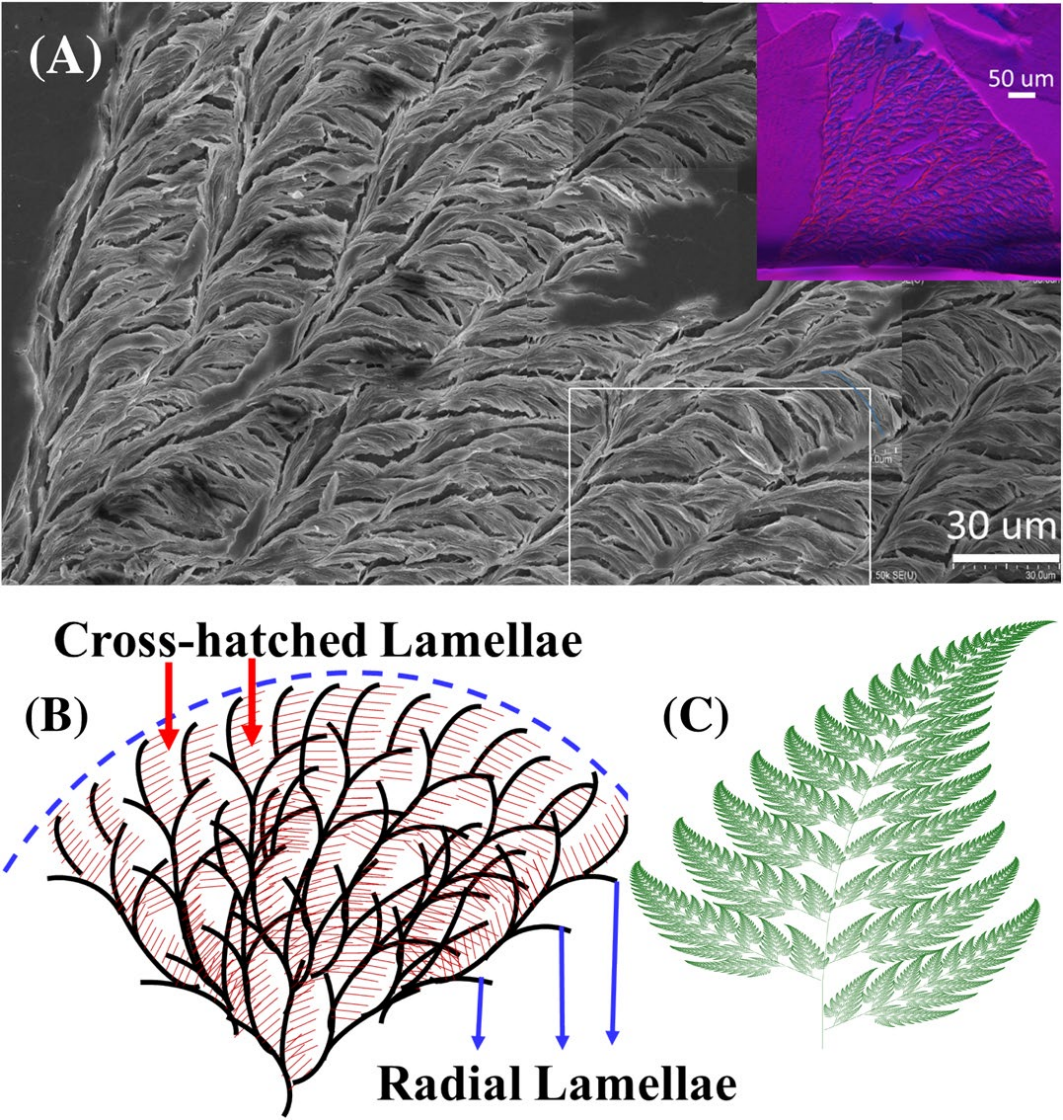
(**A**) SEM stacked-images for top surface of single-spoke fan in sector-face PLLA spherulites crystallized from PLLA/PMMA (80/20) blend at 115 °C; (**B**) scheme for fractal cross-hatch growth. Specimens after solvent-etching. (Inset: POM); and (**c**) fractal-growth fern-leaf (image taken from “Wikipedia”—open free source^25^).

Top-surface morphology of the sector-face PLLA spherulites was more carefully analyzed using AFM and SEM. Figure 8a shows SEM (stacked micrographs side-by-side to cover a complete fan-like region) for the topological images of the sector-face-morphology PLLA spherulites after acetone-vapor etching, where inset POM micrograph shows corresponding optical birefringence pattern. Several images on various zones were zip-stacked into an integral one in order to cover wider regions of the entire fan-like dendrites, which measure as wide as ~ 700–900 μm in length. The fan-like dendrites apparently grows from an original single spoke at the nucleus center, rapidly explode into hundreds or thousands of cross-hatch branches by fractal multiplication. Figure 8b illustrates fractal growth in cross-hatch patterns, starting from a single lamella, expanding quickly into fan-like splay. The growth is apparently reproduced in a fractal-growth pattern resembling a fern leaf (Fig. 8c). The fern-leaf is used here as a model analogy of the assembly of the PLLA branches. A very interesting feature is observed in the formation of the high-birefringence portion (dendritic portion) in the sector-face PLLA spherulites. The fractal divergence into a fan-like pattern of the branching lamellae appears to explode rapidly from the center part of PLLA spherulite. The dendritic face (one or two fans) is originated from a tiny hole/cavity where a single stalk of nucleating crystal rapidly re-produce and multiply into complex fan-like large aggregation.

**Figure 9:**
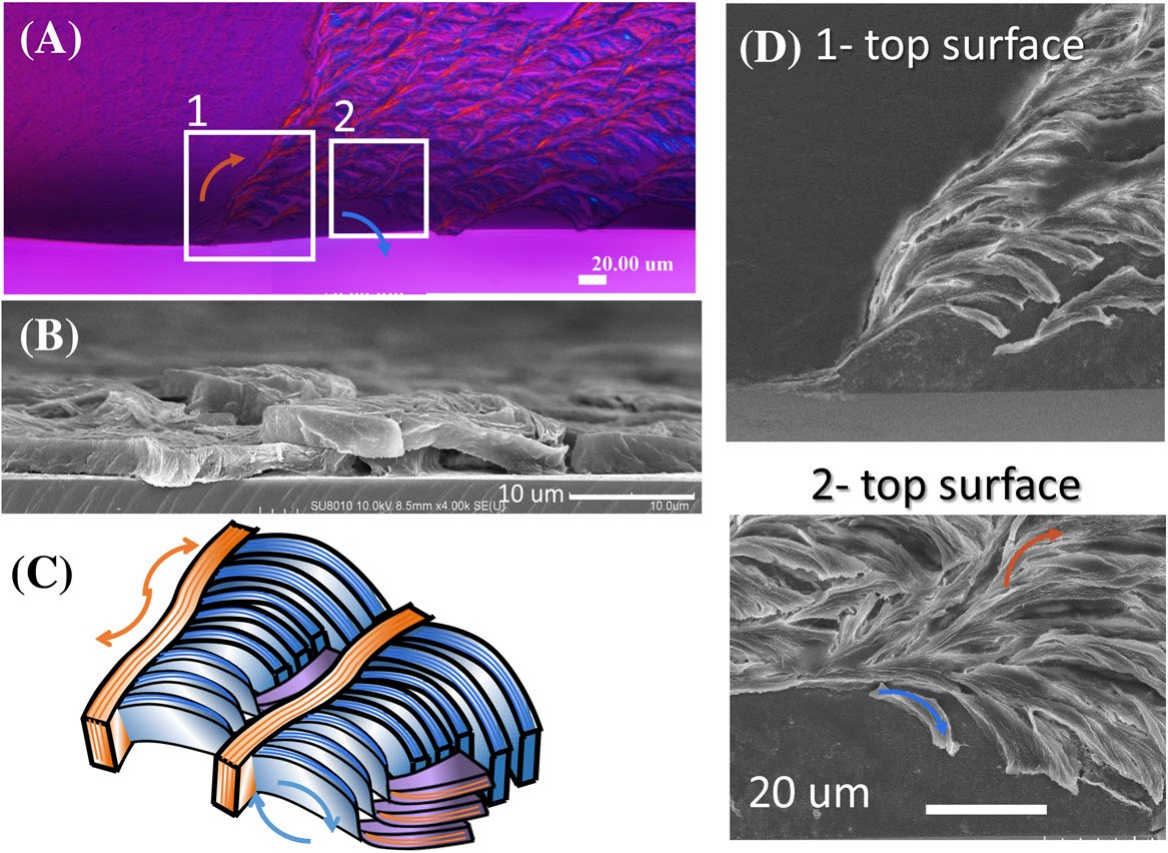
Dendrite PLLA spherulite showing three geometries of lamellae species: (**A**) POM graph showing birefringence patterns in solvent-etched sector-face PLLA spherulites, (**B**) SEM micrograph, (**C**) scheme for two dominant lamellae orientations to form cross-hatch patterns, and (**D**) top-view of radial vs. tangential lamellae in dendrites.

Further details in the PLLA dendrite were explored by directly mapping the POM micrographs with SEM images. Sector-face PLLA spherulites were first solvent-etched to remove the top-surface aggregation layer with objective to reveal better top-surface-relief features, then the specimens were characterized using POM and SEM. Figure 9 shows that in the dendrite region, three different geometries/orientations of lamellar species are spotted in the POM and SEM micrographs: orange-color birefringence corresponding to main radial edge-on lamellae plate; Blue-color birefringence corresponding to tangential branching edge-on lamellae; and magenta-color birefringence corresponding to lamellae of strictly flat-on twists, where the chain axes are parallel to the light path and lead to optical-extinction of magenta-color. Note however, flat-on lamellae with optical extinction are of minority presence, suggesting that most radial and tangential lamellae are both edge-on and they only sporadically twist into flat crystals in small fractions. Zoom-in to a lateral view (scheme in Diagram-C) exposes the lamella’s lateral width for the fibrous branches (apparently edge-on, rather than flat-on), and it reveals the lamella’s width (edge-on) being ca. 3–4 μm. The SEM micrograph (Diagram-B) also suggests that both radial main lamellae and branching fibers remain to be edge-on in the radial main stalks, but bend, twist or flip in the tangential-oriented cross-hatch branches. Furthermore, if one compares the POM with SEM characterization results on the dendritic PLLA spherulites, it is straightforward to recognize that the radial lamellae in SEM graph correspond to the blue-birefringence (with tint plates), and the cross-hatch lamellae correspond to the orange-birefringence pattern in POM. Such recognition by directly correlating the lamellae structure in the dendritic PLLA spherulites is critical, as similar optical features of lamellae assembly can also occur in circularly banded PLLA spherulites too^18^. The dendritic region (high birefringence) of the sector-face PLLA is composed of elongated platelet edge-on lamellae that are arranged almost perfectly parallel to each other and to the substrate; by contrast, the crystals in the flat region (low-birefringence eye-like region with a bivalve-shape) are nano-size fine cilia crystals aligned at various slant angles to the substrate.

**Figure 10:**
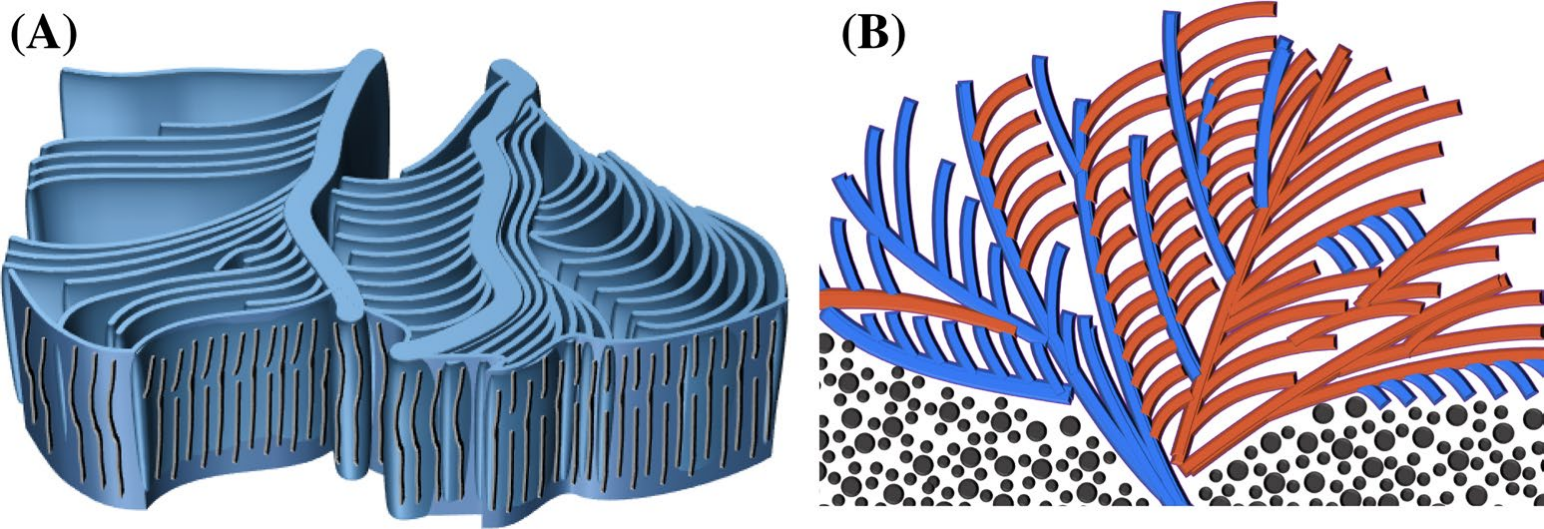
Schematics of lamellar assembly in the dendrite portion of sector-face PLLA spherulite: (**A**) top view and lateral view, and (**B**) optical birefringence pattern correlating with lamellar assembly and orientations.

Figure 10 shows 3D schematics of lamellar assembly in the dendrite portion of sector-face PLLA spherulite from (a) top and lateral views, and (b) corresponding optical birefringence. The radial main lamellae are thicker and edge-on oriented; yet the branching lamellae are also edge-on oriented but thinner in thickness (≈ 300 nm) and narrower in width. Note here that the thinner fibrous lamellae, with lower height profiles, in the valley region (tangentially oriented), are not necessarily twist into flat-on; they simply are thinner/narrower branches, but still remain as edge-on (i.e., lamellae plates standing perpendicular to the substrate), evolving as branches from the main radial lamellae and the branching point distance is 35 μm. That is to say, the alternate optical blue/orange birefringence stripes (viewed with tint plates) in POM micrographs are not due to continuous edge-on/flat-on lamellae alternating twists commonly viewed as a responsible mechanism in the literature. The alternate birefringence stripes or rings are actually due to periodic branching from radial-direction to tangential-direction, as well as periodic thickness/width variation in lamellae plates. The periodic direction changes by branching are the main factor for striped or ringed birefringence patterns viewed in POM. The general 3D structure shows a grating assembly composed of thicker/wider radial lamellae (ca. *t* ≈ 3 μm) (edge-on in radial direction) intersecting with thinner/narrower cross-hatch lamellae (ca. *t* ≈ 300 nm) (in tangential direction).

**Figure 11:**
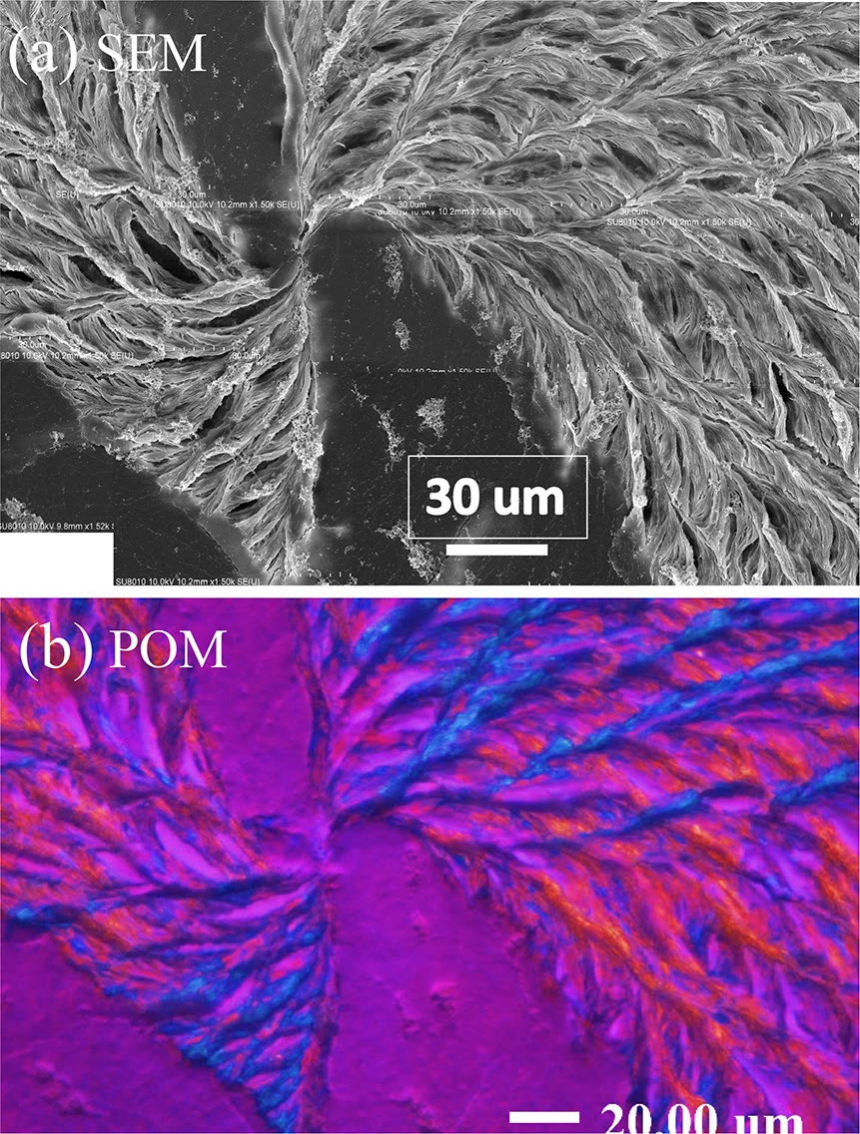
Top-surface topography of dual-fan sector-face PLLA spherulites crystallized from PLLA/PMMA (80/20) blend at 115 °C after solvent-etching: (**a**) SEM, and (**b**) POM micrograph (flipped to mirror image), showing lamellae in two opposite directions of nucleus center.

Origin of the sector-face PLLA spherulites appears to be puzzling, which requires further investigation. Figure 11 shows (a) SEM top-surface topography, and (b) POM micrograph of dual-morphology sector-face PLLA spherulites crystallized from PLLA/PMMA (80/20) blend at T_c_ = 115 °C (specimens after solvent-etching). The POM micrograph is flipped to its mirror image, as the CCD’s (i.e., charge-coupled device for image recording/processing) of POM and SEM are mirror images to each other; thus, for direct and convenient comparison between the SEM and POM images, the POM micrograph is flipped to its mirror-image. One can see that in this PLLA spherulite, the dendritic face of high optical-birefringence emerges from both sides (i.e., two original spokes emerging in two opposite directions from a common nucleus center) to finally fill most of the spherulites, leaving only narrow twin eye-like regions of low optical birefringence. That is to say, if the dendritic lamellae emerge out in one direction to fill up only one fan (from one nucleus hole), rather than growing in both sides to two fans, then a sector-face PLLA spherulite is resulted. Thus, the mechanism of formation of the sector-face PLLA spherulites, along with two other types of PLLA spherulites, can be proposed. Statistically and kinetically, the growth rate and directions of the dendritic lamellae to evolve from the cavity hole of the nucleus region lead to three possibilities of spherulite morphology: (1) entirely low-birefringence PLLA spherulites (no dendritic lamellae), (2) entirely high birefringence dendritic spherulites (dendrites filling up the entire spherulite in two opposite directions, except for the narrow twin eye-like region), and (3) half-and-half low-birefringence and high-birefringence lamellae (dendrites filling in explosion fractal pattern in one direction only). Note that all lamellar branches bend synchronizingly all in the clockwise direction: those on right-hand-side bend to convex-down-curve shapes and those on left-hand-side bend to concave-up-curve shapes^26^, with a nearly mirror-image symmetry.

Not only the polymeric sector-face PLLA spherulites in PLLA/PMMA blends display such unusual curvature-bent dendritic cross-hatch patterns, but also many small-molecule spherulites do, as shown in phthalic acid (PA) crystallized in PA/tannic acid (TA) mixtures, showing ring-banded aggregates^27^. Similarly, PA grown and crystallized in PA/poly(ethylene oxide) (PEO) mixtures also display periodically ring-banded aggregates packed with fractal-branching grate structures where each of the fractal units contains two entities: main stalks (as the ridge band) of discrete crystalline aggregations arranged along a radial direction, and fern-like dendrites (as the valley band) arranged along a tangential direction^28^. Wolf et al.^29^, by using POM and AFM analyses, also recently reported a photo-polymerization strategy with simultaneous cross-linking and crystallization of polysiloxane chains into millimeter-sized leaf-like polycrystalline structures, where all the “leaves” synchronizingly bend in the clockwise curvature (as shown in POM images) that is similar or almost identical to the feature of the branching-leaves in the sector-face PLLA spherulites. These facts demonstrate that the fractal growth of branches evolving as cauliflower-like patterns is a quite universal mechanism, regardless of polymeric or small-molecule compounds, either as radially grown dendrites or circularly ring-banded aggregates in spherulites. That is, periodicity (optically and morphologically) commonly present in spherulitic aggregates is proposed to arise more often from sudden discontinuous branching than that from a helical twist of the growing single-stalk crystal. Nevertheless, occasional twists, in transition from a compact space to less-crowded one, may certainly also occur as the branches grow and evolve from the main stalks; however, continuous helix-twist of a single-stalk crystal, from the nucleus center to periphery, is not likely as it will not be able to fill the ever-expanding space increasing radially.

**Figure 12:**
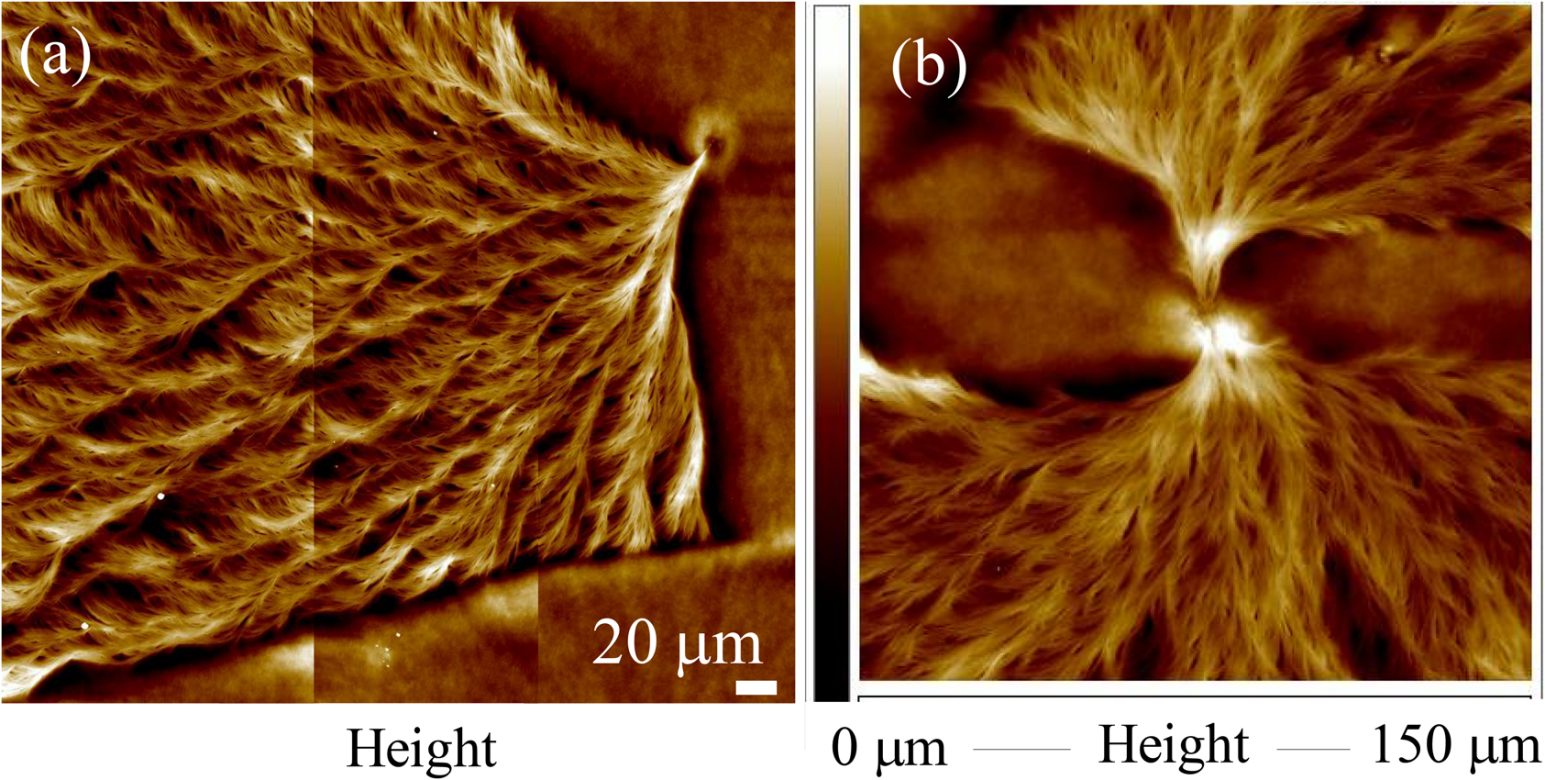
AFM height images for (**a**) single-spoke fan vs. (**b**) three-spoke fans in sector-face PLLA.

Figure 12 shows AFM height images for (a) single-spoke fan vs. (b) four-spoke or multiple-spoke fans in the sector-face PLLA spherulites in specimens after acetone-etching. Packing mechanisms of the dendritic face in the sector-face PLLA spherulites can be summarized as following. Originating from a common nucleus center, there are needle-like spokes of lamellae (two in each of two directions) evolving from the center hole of nucleus; the needle-like spokes then explosively fan out with fractal growth outwards into four fan-like lamellar canopies. As the two lamellar fans in one direction tend to merge or overlap into one with each other, there are usually two fans (one in each of two direction) left when fully grown. The nanometer-size (ca. ~ 100 nm in width or diameter) sheaf-like lamellae in the nuclei center rapidly grow with an explosive expansion into fan-like dendrites near the nucleation-center region, and these dendrites are actually made of nanometer cilia crystals growing with fractal-repetition branches. These initially nanometer cilia lamellae near the nuclei finally thicken, merge, and reach micrometer-size bundles (ca. ≈ 3) as they grow further outwards to midway and periphery of the sector-face PLLA spherulites, as shown earlier in Fig. 8 SEM micrographs. Interestingly, such explosive fractal growth of crystals (in AFM images of Fig. 12a,b) from a tiny nucleus stem to massive fan-like “ballistic” expansion can be drawn to resemble a botanic cauliflower, which develops from a single stem on the ground rapidly to a complete full-body semi-spherical head covered with thousands of little white flower buds in just 5–6 fractal-growth cycles. That is, the unique growth of thousands of PLLA lamellae evolving from a single stem (from the nucleus cavity) is properly termed as “ballistic” patterns with a fan-like cross-hatch network. Note that the growth can be one-armed into one fan or two-armed into two fans with approximate symmetry. Ballistic patterns growing from a single nucleus seed were considered by Ferreira et al.^30^, where they mathematically treated crystal growth into ballistic fractal and morphological transition between diffusion-limited and ballistic aggregation growth patterns.

### Branching by Fibonacci sequence in random fractal patterns

**Figure 13:**
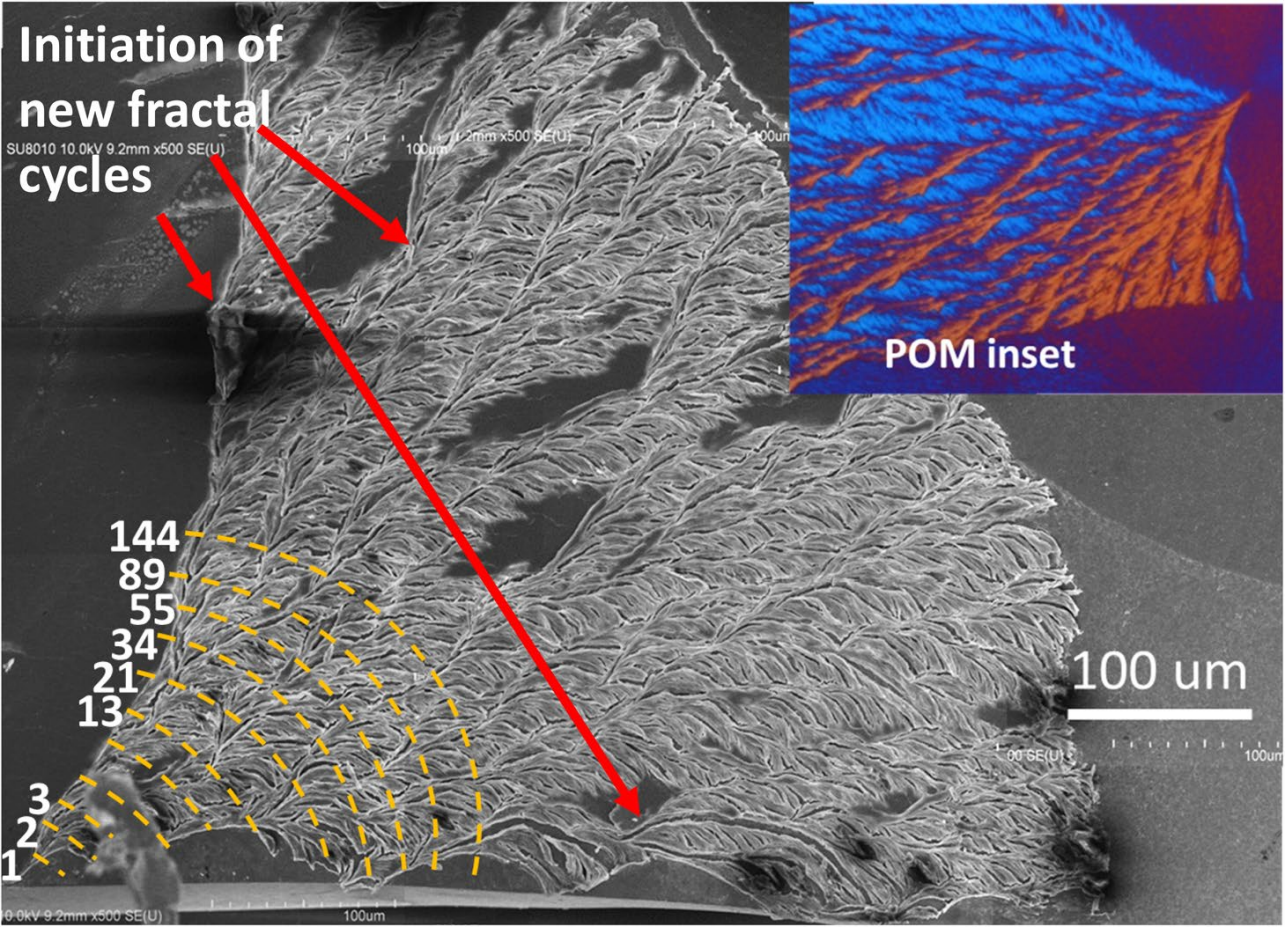
Stacked SEM micrographs (by zip-stacking several individual micrographs side-by-side to an integral mega-graph) for complete view of a single explosive sector in the sector-face PLLA spherulites crystallized from PLLA/PMMA (80/20) blend crystallized at 115 °C after solvent-etching, yellow dashed-lines indicate lamellar fractal-branching cycles. Inset POM (flipped to its mirror image) on upper-right corner.

In order to view the expansive region of the entire explosive cauliflower-like pattern of the dendritic lamellae that originally evolve by fractal repetitions from a single strand of fiber-like lamellae, a special SEM technique was utilized. Many separate SEM characterizations were first performed on discrete spots of the crystallized specimens; the separate micrographs were then stacked into an integral one. The dendritic face of the sector-face PLLA spherulites as analyzed using SEM at higher magnifications can be more vividly viewed in wider dimensions by stacked several separate SEM micrographs. Figure 13 shows a stacked SEM micrograph (by “zipping together” several discrete SEM micrographs to cover continuous wider regions) for a massively explosive sector in the sector-face PLLA spherulites. The fractal tree-branch patterns in the dendritic face of PLLA sector-face spherulites are quite apparent. Thicker radial-oriented lamellae (ca. ≈ 3 μm) are seen to intersect with thinner tangential branching lamellae (ca.  ≈ 300 nm) (i.e., cross-hatch lamellae). The intersection angle between the radial and tangential lamellae is initially at 60°; however, the tangential lamellae further bend from the main stack and form perpendicular orientation with the radial lamellae. Note all tangential lamellae, once evolving from the radial ridge lamellae with the radius of curvature being 13 μm, collectively and consistently bend in the same clockwise direction without exception. AFM characterization results were used to support the proof of such an unusual morphology. Note that it is evidently apparent that the massive cauliflower-like sector (packed of high-birefringence self-assembled lamellae) originates from a single tiny stalk (less than 1 μm) at the nucleus center (located at lower-left corner of SEM micrograph).

**Figure 14:**
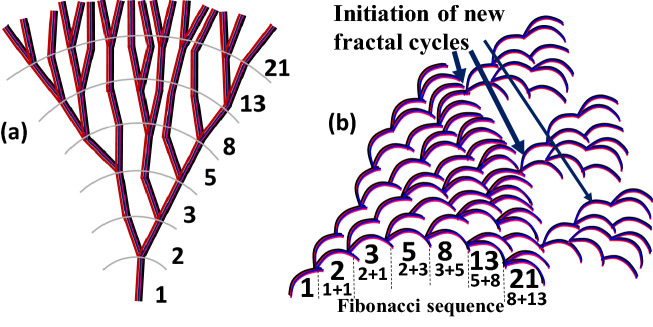
Single-spoke fan in sector-face PLLA spherulites in explosive outward growth based on (**a**) Fibonacci-sequence branching, (**b**) cycles of branching in repetitive growth from front sides of matured periphery.

The Fig. 14a shows that the number of branches in the fan-like lamellar aggregates increases explosively or “ballistically” with increasing radial distance from the original nucleus center outward to the periphery, roughly following the Fibonacci sequence that is commonly seen in many nature’s growths with fractal patterns. Furthermore, the explosive growth may not always continue all the way from the nucleus to the periphery owing to periodic drainage of the diffusing molten species. In those cases, new cycles of branching can be re-initiated again in repetitive growth from the front sides of a matured periphery, as shown in Fig. 14b. Then, new fan-like dendrites by Fibonacci-sequence fractal growth re-start from the matured periphery till drainage of all available molten species.

Branching is widely found in either ringless, ring-banded, or dendritic spherulites of polymers or small-molecule compounds alike. Branching lamellae (from a mother crystal) are secondary-crystallized according to the classical crystal growth mechanisms, which is growth by stepwise attachment to a primary crystal species. Crystal branching in aggregation into final spherulites is reviewed in a recent report by Kniep et al*.*^31,32^ to discuss the diverse phenomena of crystal branching in spherulites, where one of many possible factors is presence of amorphous diluents in the crystalline species, providing dilution or mutual interactions. In addition, Shtukenberg et al*.*^33,34^ suggested that the internal stress in crystal growth is the most important factor for crystal branching in spherulite. In crystal interfaces, bending, scrolling, or twisting, etc., there may be some sources of internal stresses that can be induced to spawn more branching lamellae in a spherulite. Introduction of the amorphous PMMA diluent (in the PLLA/aPMMA blend) has a profound effect on setting the unusual sector-face PLLA spherulites, as well as explosive dendritic branching. With the amorphous polymeric diluent (aPMMA) along with non-crystallizing PLLA chains periodically being expelled to the crystal fronts, the nucleation sites in each end of growth cycles are proportionally increased, leading to favorable growth of branches increasing explosively in approximate Fibonacci sequence in cycling growth.

## Conclusion

Peculiar “sector-face” PLLA spherulites (at T_c_ = 115–120 °C and only appearing in PLLA/diluent systems), with unusually high birefringence in one face being inter-sectored by dot-like crystals of extremely low birefringence in the other face, has been analyzed for detailed mechanisms. AFM on top surfaces and SEM analysis by interior dissection, coupled with selective acetone-vapor-etching on specimen, in the interiors of the sector-face PLLA spherulites has revealed striking evidence for assembly mechanism via explosive fractal growth from the nucleus center to one or two fan-like sectors packed with mostly edge-on fibrous lamellae. Furthermore, the number of lamellar branches increases approximately in the Fibonacci sequence (1, 2, 3, 5, 13, 21, … 55, … etc.), which is in line with the many well-known growth patterns commonly seen in the nature. Certainly, the nature’s order in crystal assembly may not be as rigorously exact as what described by a mathematic sequence, because the lamellae are not discretely distinguishable from each other and the number may be difficult to count as there may be occasional overlaps and hidden or jammed lamellae. Biological structures can evolve through million-year adaptation to get closer to an ideal form to achieve specific functions; by comparison, crystal assembly is done in a flash time frame with no chance to re-adjust at all. Nevertheless, the explosive growth of the lamellae from a nucleus center in the PLLA dendrites can be approximated by the Fibonacci sequence, more or less in random-fractal similarity.

The periodic branching growth of lamellae in the sector-face PLLA spherulites can be biomimetic to a cauliflower pattern. The fractal growth in the dendritic sector is characterized with periodic repetitive growth of multiple branches, which resembles a botanic cauliflower with full-body semi-spherical head covered with thousands of little white flower buds in just 5–6 fractal repetitions. As the main stalks and branches naturally intersect at an oblique angle of 60°–90° (balanced by the crystal lattice faces and mutual branching crowdedness), the assembled lamellae in the highly birefringent PLLA fan-like sector display alternate blue/orange optical stripes (in POM with tint plates), which are commonly seen in assembled lamellae of spherulites regardless of radially dendritic or circumferentially circular ringed crystalline entities.

## Methods

### Materials and procedures

Poly(l-lactic acid) (PLLA) of 11,000 g/mol weight-average molecular weight (M_w_) was used (Polysciences, Inc.). Atactic poly(methyl methacrylate) (aPMMA) of M_w_ = 240,000 g/mol (PMMA-240 k), PDI = 1.49 (Aldrich) was used as the main diluent to blend with PLLA. Weight-average molecular weights (M_w_), polydispersity index (PDI), glass transition temperatures (T_g_), as well as melting peak temperature (T_m_) of each material used in this study are listed in Table 1. Different compositions of 2 and 4 wt% PLLA/aPMMA (80/20) blend solutions were prepared by solvent blending at 50 °C using chloroform as the solvent. The mixture solutions were then cast on glass substrates (on a hot plate preset at 50 °C) to make thin-film or thick-bulk samples. Different thicknesses of film samples (varying from 5 to 30 µm) were obtained by controlling the number of solution casting on the substrate. Degassing process was performed at 40 °C in a vacuum oven for more than 4 h to get rid all residual solvent properly. T_c_ of crystallization was set at 115–120 °C. Molecular weight of either PLLA or PMMA has been proven to influence the spherulite’s morphology. In this work, the molecular weight of PLLA was fixed at (designated as “11 k-PLLA”; while that of PMMA is fixed at 240 k (designated as “240 k-PMMA”. When the number designation is omitted from codes, PLLA and PMMA in the work all refer to these two M_w_’s.

**Table 1:** Materials used and their physical properties.

Materials	Source	M_w_ (g/mol)	PDI	T_g_ (°C)	T_m_ (°C)
PLLA	Polysciences, Inc.	11,000	1.1	45.3	155
aPMMA	Aldrich Co.	240,000	1.49	99	–

Mixing PLLA with polymeric diluents was performed by solvent-blending, followed with proper drying and de-gassing. A special solvent-etching technique (acetone vapor exposure for controlled time) was used for enhancing contrast of crystal morphology in SEM analysis. After designated crystallization at specific T_c_’s, the PLLA/aPMMA film samples were fractured so that the interior lamellae, with proper etching off the amorphous constituent, could be observed from all possible perspective angles, which all together provided collective views to construct 3D lamellae assembly in the complex spherulites. Film thickness was kept at 5 μm. The fractured samples were then exposed to acetone vapor on a 50 °C preset hotplate for 20 min, then etched/washed by immersing the samples in acetone liquid for 2 s to disclose the detailed lamellar crystal structure in the spherulites. Acetone etching on specimens was used to enhance the contrast in sector-face PLLA spherulites. Degassing was performed after etching in a 40 °C vacuum oven for a couple of hours. For interior crystal/lamellae analyses, the specific T_c_-crystallized PLLA/aPMMA (80/20) spherulites was carefully fractured across the film samples attached on glass substrate. Diamond knife was used to pre-cut the glass slides for directing the fracture lines along intended spots.

### Apparatus

*Light-polarized optical microscope *(POM, Nikon Optiphot-2), equipped with a Nikon Digital camera system for microscopy Digital Sight (DS-U1) and a programmed microscope hot stage (Linkam THMS-600 with T95 temperature programmer) was used to observe the spherulitic development and morphology during crystallization, prior, and after etching treatment.

*Atomic-force microscopy* (AFM, diCaliber, Bruker-Veeco Co., Santa Barbara, CA) was used in an intermittent tapping mode with a silicon tip (*f* = 300 kHz, *r* = 10) installed to scan the surface topography of specimens.

*Scanning electron microscopy (SEM)* Top-surface relief patterns and interior observations were analyzed using field-emission scanning electron microscopy (FE-SEM, HITACHI SU8010) on both virgin and etched samples. Gold sputtering was performed on the crystallized samples prior to SEM characterization.

*Differential scanning calorimeter* (DSC Diamond, Perkin-Elmer Corp., USA), equipped with an intra-cooler, was used to measured thermal transition behavior, or thermal treatments of samples. During the scanning or thermal treatments of samples, a continuous flow rate of nitrogen in the DSC chamber was maintained to prevent sample degradation. Instrument was duly calibrated with standards.

Scanning electron microscopy (SEM, Model: FEI Quanta-400F) was used to characterize the detail lamellar arrangement of fractured surfaces of samples in correlation to their top surfaces before and after the etching process. The samples were coated with gold vapor deposition using vacuum sputtering before the SEM characterization.

*Small-angle X-ray scattering* (SAXS, Bruker Gmbh, Karlsruhe, Germany) measurements (1D and 2D) were performed at the beamline *IμS*. X-ray radiation source of energy of 50 kV and a detector VANTEC-2000 were used to collect 2D SAXS patterns. The distance from the sample to the detector was 3,542 mm. The scattering vector, *q* (*q* = 4π/*λ* sin *θ*), with scattering angle *θ*, in these patterns, was calibrated with silver behenate. After background subtraction and data reduction, 1D SAXS profiles were obtained with relative intensity (*Iq*^*2*^) distributions as a function of *q*.

## Supplementary information

Supplementary information.
