# Supplementary material for: Explosive Fibonacci-sequence growth into unusual sector-face morphology in poly(l-lactic acid) crystallized with polymeric diluents
Source: Sci Rep. 2020 Jul 2;10:10811. doi: 10.1038/s41598-020-67567-5 (PMC7331819; doi:10.1038/s41598-020-67567-5)
Supplement: Supplementary file 1 — Supplementary information. [file 41598_2020_67567_MOESM1_ESM.docx]

**Supporting Information**

**Explosive Fibonacci-Sequence Growth into Unusual Sector-Face Morphology in Poly(L-lactic acid) Crystallized with Polymeric Diluents**

Graecia Lugito^a,b^, Selvaraj Nagarajan^a^, and Eamor M. Woo*^a^,

^a^Department of Chemical Engineering, National Cheng Kung University,

No. 1 University Road, Tainan 70101, Taiwan.

^b^Department of Chemical Engineering, Institute Technology Bandung,

Jl. Ganesha 10, Bandung 40132, Indonesia.

**Supporting Info. Table S1:** Long-period and lamellar parameters in PLLA/PMMA crystallized at different T_c_’s_._ (where L_o_-long period, L_a_-amorphous thickness, L_c_-crystal thickness).

**1D-electron density correlation function (K(z)):**

The PLLA lamellar thickness in PLLA spherulites crystallized from PLLA/PMMA (80/20) blend with different T_c_ was estimated from the SAXS curves. Normalized 1D-electron density correlation funstion (K(z)) is below:

K(z) = $\frac{1}{Q}\int_{0}^{\alpha} q^{2} I\left( q \right)\cos\left( qz \right)dq$

Where Q = $\int_{0}^{\alpha} q^{2}I\left( q \right)dq$, z is co-ordinate distribution electron density, q- scattering vector, I(q) is intensity.

**Table S1:** Long-period and lamellar parameters in PLLA/PMMA (80/20) crystallized at various T_c_’s_._ (where L_o_-long period, L_a_-amorphous thickness, L_c_-crystal thickness).

| T_c_ (^o^C) | Q(nm^-1^) | L_o_ (nm) | L_a_ (nm) | L_c_ (nm) |
| --- | --- | --- | --- | --- |
| 80 | 0.340 | 15.5 | 6.6 | 8.9 |
| 100 | 0.360 | 15.4 | 6.2 | 9.2 |
| 120 | 0.438 | 13.3 | 5.3 | 8.0 |
| 125 | 0.432 | 13.9 | 5.7 | 8.2 |
| 130 | 0.432 | 13.9 | 5.7 | 8.2 |
